# Derivation of the first clinical diagnostic models for dehydration severity in patients over five years with acute diarrhea

**DOI:** 10.1371/journal.pntd.0009266

**Published:** 2021-03-10

**Authors:** Adam C. Levine, Meagan A. Barry, Monique Gainey, Sabiha Nasrin, Kexin Qu, Christopher H. Schmid, Eric J. Nelson, Stephanie C. Garbern, Mahmuda Monjory, Rochelle Rosen, Nur H. Alam

**Affiliations:** 1 Department of Emergency Medicine, Warren Alpert Medical School, Brown University, Providence, Rhode Island, United States of America; 2 Rhode Island Hospital, Providence, Rhode Island, United States of America; 3 International Centre for Diarrhoeal Disease Research, Bangladesh (icddr,b), Dhaka, Bangladesh; 4 Department of Biostatistics, School of Public Health, Brown University, Providence, Rhode Island, United States of America; 5 Emerging Pathogens Institute, University of Florida, Gainesville, Florida, United States of America; 6 Department of Behavioral and Social Sciences, School of Public Health, Brown University, Providence, Rhode Island, United States of America; University of Washington, UNITED STATES

## Abstract

Diarrheal diseases lead to an estimated 1.3 million deaths each year, with the majority of those deaths occurring in patients over five years of age. As the severity of diarrheal disease can vary widely, accurately assessing dehydration status remains the most critical step in acute diarrhea management. The objective of this study is to empirically derive clinical diagnostic models for assessing dehydration severity in patients over five years with acute diarrhea in low resource settings. We enrolled a random sample of patients over five years with acute diarrhea presenting to the icddr,b Dhaka Hospital. Two blinded nurses independently assessed patients for symptoms/signs of dehydration on arrival. Afterward, consecutive weights were obtained to determine the percent weight change with rehydration, our criterion standard for dehydration severity. Full and simplified ordinal logistic regression models were derived to predict the outcome of none (<3%), some (3–9%), or severe (>9%) dehydration. The reliability and accuracy of each model were assessed. Bootstrapping was used to correct for over-optimism and compare each model’s performance to the current World Health Organization (WHO) algorithm. 2,172 patients were enrolled, of which 2,139 (98.5%) had complete data for analysis. The Inter-Class Correlation Coefficient (reliability) was 0.90 (95% CI = 0.87, 0.91) for the full model and 0.82 (95% CI = 0.77, 0.86) for the simplified model. The area under the Receiver-Operator Characteristic curve (accuracy) for severe dehydration was 0.79 (95% CI: 0.76–0.82) for the full model and 0.73 (95% CI: 0.70, 0.76) for the simplified model. The accuracy for both the full and simplified models were significantly better than the WHO algorithm (p<0.001). This is the first study to empirically derive clinical diagnostic models for dehydration severity in patients over five years. Once prospectively validated, the models may improve management of patients with acute diarrhea in low resource settings.

## Author summary

More than a million adults and older children die each year from diarrhea, with the vast majority living in low- and middle-income countries. Accurately assessing hydration status remains the most critical step in caring for these patients. While most patients with diarrhea can be managed safely at home, a small proportion will have severe dehydration and require intravenous fluid in a hospital setting to prevent organ damage and death. Despite dramatic improvements in diarrhea care around the world over the past several decades, there are still no evidence-based tools that doctors and nurses in low resource settings can use to determine an adult patient’s hydration status. Our study collected data on more than two thousand patients with acute diarrhea over the age of five years at Dhaka Hospital in Bangladesh. Using artificial intelligence techniques, we developed two new clinical diagnostic tools that can accurately and reliably predict the severity of dehydration in patients with diarrhea, which we will incorporate into a simple mobile phone application for use by doctors and nurses in low resource settings. Once validated, these new tools could improve care for patients with acute diarrhea worldwide.

## Introduction

Despite significant reductions in mortality over the past several decades, diarrheal diseases remain the 5^th^ leading cause of years of life lost globally, accounting for over 1.5 million deaths in 2017, including over one million deaths in adults and children over five years [1,2]. The vast majority of diarrheal episodes follow a relatively benign course; however, approximately 5% of cases in adults and older children lead to moderate or severe disease requiring advanced medical management [3]. The elderly may be especially susceptible, with patients over age 50 accounting for more than half of diarrheal deaths in 2017 [1,2,4–6].

As the severity of dehydration from acute diarrhea varies widely among patients, accurately assessing hydration status remains the most critical step in providing appropriate treatment and reducing morbidity and mortality [7–11]. Patients with severe dehydration require immediate resuscitation with intravenous fluids to prevent hemodynamic compromise, organ ischemia, and death, while those with mild to moderate dehydration can be safely treated with oral rehydration solution (ORS) alone [12]. By ensuring that ORS is used for the treatment of appropriate patients rather than more costly intravenous fluids, accurate assessment of dehydration status can also improve the cost effectiveness and quality of care, reducing both inpatient hospitalizations and adverse events [12].

**Fig 1:**
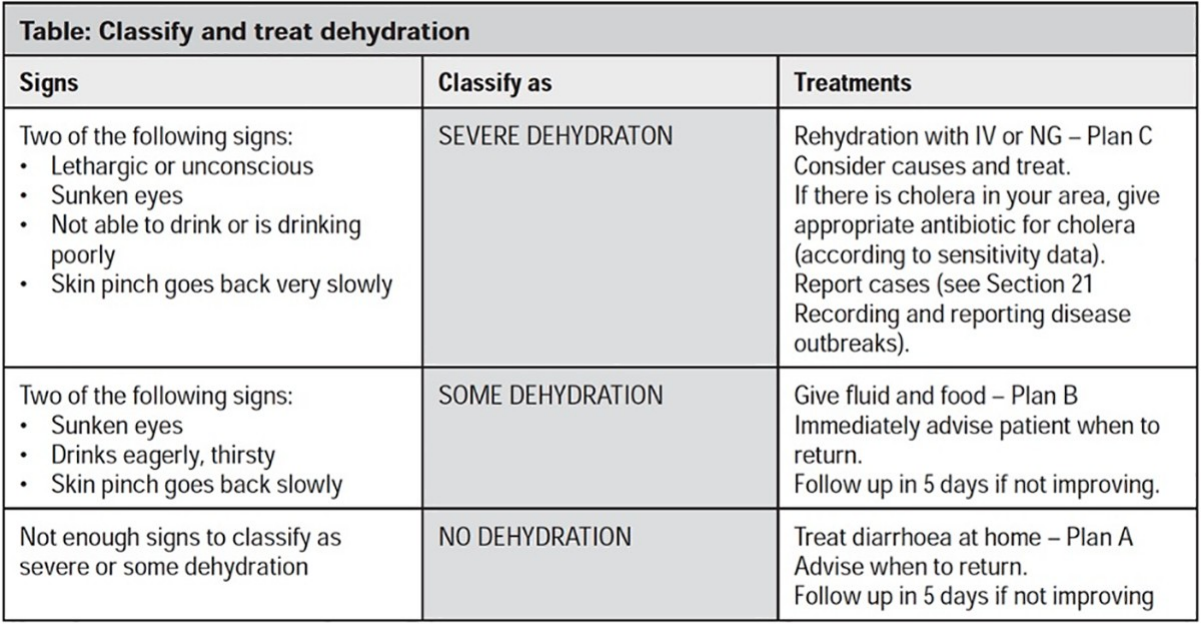
WHO IMAI Algorithm for Dehydration Assessment in Patients with Acute Diarrhea [10,13].

Currently, the World Health Organization (WHO) Integrated Management of Adolescent and Adult Illness (IMAI) guidelines recommend a simple algorithm for determining the severity of dehydration in adolescents/adults with acute diarrhea, based on a similar WHO Integrated Management of Childhood Illness (IMCI) algorithm developed for children under five years (Fig 1: WHO IMAI Algorithm for Dehydration Assessment in Patients with Acute Diarrhea) [10,13]. Two additional clinical diagnostic models have been empirically derived and validated for the assessment of dehydration in children under five years with acute diarrhea: the Clinical Dehydration Scale (CDS) in North America and the Dehydration: Assessing Kids Accurately (DHAKA) score in Bangladesh [11,14–16]. While several studies have assessed the accuracy of the WHO, CDS and DHAKA models in different contexts, none have been validated for the assessment of dehydration in patients over five years with acute diarrhea [17–20]. Differences in both adult physiology and diarrhea etiology may compromise the accuracy of clinical diagnostic models originally developed for use in young children [21–23].

The primary aim of this study is to empirically derive clinical diagnostic models for dehydration severity in patients over five years with acute diarrhea in order to support clinicians in the initial triage and management of these patients, especially in low resource settings.

## Methods

### Ethics statement

Ethical approval for the study was granted by the International Centre for Diarrhoeal Disease Research, Bangladesh (icddr,b)’s Ethical Review Committee (PR-18077) and the Rhode Island Hospital Institutional Review Board (1244580). Formal verbal/written consent was obtained from each participant and/or their parent/guardian if under 18 years old in the native language, Bangla.

### Study design

Data were collected as part of the Novel, Innovative Research for Understanding Dehydration in Adults and Kids (NIRUDAK, meaning “dehydrated” in Bangla), a prospective cohort study of patients over five years presenting with diarrhea to the International Centre for Diarrhoeal Disease Research, Bangladesh (icddr,b) Dhaka Hospital in Bangladesh between March 2019 and March 2020. Icddr,b is an internationally renowned diarrheal research center that provides free clinical services to a catchment area of over 17 million people in Dhaka and the surrounding area [24].

### Study setting and population

Study staff randomly selected patients for screening on arrival 24 hours per day, 7 days per week at icddrb’s Dhaka Hospital rehydration unit. Random selection was accomplished through the use of a black pouch filled with white and colored marbles, in which study staff drew a marble from the pouch each time a patient presented to the rehydration unit. Patients were selected for screening if a colored marble had been chosen. Selected patients were excluded if they were enrolled previously in the study, had less than 3 loose stools in 24 hours, diarrhea lasting longer than 7 days, or an initial diagnosis by the triage physician other than gastroenteritis (such as sepsis, systemic viral infections, hepatitis, pancreatitis, or appendicitis). For eligible patients, research staff provided the patient and/or their parent/guardian with information about the goals, risks, and benefits of the study and obtained verbal/written consent in Bangla.

### Staff training and oversight

Local general practice nurses with at least two years of clinical experience were hired outside of the icddr,b clinical nursing pool to collect data for this study. Prior to the start of the study, research staff received one week of didactic and hands-on training in all study procedures, including the assessment of clinical symptoms/signs of dehydration. Specific details related to assessments of clinical symptoms/signs of dehydration can be found in the first section of the appendix (S1 Text). To ensure quality of data collection, study staff conducted random, unannounced observations of patient enrollment and clinical assessments throughout the study. These random observations occurred after every 50 enrollments for the first 100 enrollments. If no significant concerns were raised, the observations were reduced in frequency to every 100 patient enrollments thereafter. In addition, double data entry was utilized for all study data in order to reduce the likelihood of errors related to data entry.

### Study procedures

After informed consent, patients were immediately weighed to the nearest 0.1 kilograms using an electronic Seca 952 chair or Seca 984 bed scale. Patients were then independently assessed by two research nurses, blinded to each other’s clinical assessments, for 9 basic symptoms/signs of dehydration, including mental status, thirst, skin pinch, eye level, mucous membranes, respiration depth, radial pulse, capillary refill, and urine output, as well as 7 additional symptoms/signs including number of vomiting episodes within 24 hours of presentation, number of diarrheal episodes within 24 hours of presentation, diarrhea duration at presentation, heart rate, systolic blood pressure, diastolic blood pressure, and mid-upper arm circumference (MUAC), which were chosen *a priori* based on a review of the literature and consultation with expert clinicians at icddr,b (S1 Text) [25–30]. Social and demographic information were obtained afterward from either the patient or parent/guardian.

After this initial assessment, all patients were managed according to standard icddr,b protocols. In addition, patients were weighed every 4 hours on the same scale to determine their post-hydration stable weight. Those who did not achieve a stable weight prior to discharge were called daily for up to 10 days or until their diarrhea resolved, then asked to return for a final weight check.

### Laboratory methods

Two stool samples of at least 2 mL per vial were collected from each patient—one for bacterial culture and molecular (PCR) testing and one for storage in 70% ethanol. Isolation and identification of stool samples were performed using standard procedures [31]. Salmonella spp. and Shigella spp. were isolated by growth on MacConkey agar and Salmonella-Shigella agar with enrichment in Selenite F broth followed by antisera panel testing (Denka Seiken, Tokyo, Japan). V. cholerae was isolated by growth on tellurite taurocholate gelatin agar (TTGA) media with enrichment in Bile Peptone broth. Campylobacter spp. were isolated by growth on Brucella agar, and Aeromonas spp. were isolated by growth on TTGA and gelatin agar followed by phenotypic characterization of long-sugar metabolism. Susceptibility to antimicrobials was determined by the Kirby-Bauer standard disc diffusion method on Muller–Hinton agar with commercial discs, and the results were reported as sensitive, intermediate, and resistant by a method based on the cutoff of the zone size for different antibiotics according to the latest available Clinical and Laboratory Standards Institute guidelines [32].

E. coli strains were isolated by growth on MacConkey agar followed by purification of bacterial DNA via the boiling method. After preparation and completing the PCR assay, the samples were identified using gel electrophoresis and results were reported as positive or negative by a method based on comparison of the target band of the unknown sample with that of a control.

### Data analysis

#### Analysis of nutritional status

Patients between the ages of 5 to 9 years old were categorized as severe wasting if MUAC measurement was < 135 mm, moderate wasting if MUAC measurement was > 135 mm to < 145 mm and normal if MUAC measurement was > 145 mm. Patients between the ages of 10 to 14 years old were categorized as severe wasting if MUAC measurement was < 160 mm, moderate wasting if MUAC measurement was > 160 mm to < 185 mm and normal if MUAC measurement was > 185 mm. For patients 15 years of age and older, severe wasting was defined as a MUAC measurement < 185 mm, and severe wasting was categorized as a MUAC > 185 mm to < 210 mm. A patient 15 years and older was characterized as being normal if they had a MUAC measurement > 210 mm [33,34].

#### Analysis of outcome

Percent weight change with rehydration was used as the criterion standard for percent dehydration in our study, as recommended in the literature [25,35–37]. As patients were rehydrated, their weight increased until they become euvolemic and their kidneys begin to diurese excess fluid, at which point their weight stabilized. For each patient enrolled, the two highest consecutive weight measurements that differed by less than 2% were averaged to determine their stable weight, which was used as their post-illness weight [38]. For patients who did not reach a stable weight prior to discharge, their return weight after symptoms resolved was used as their post-illness weight. Percent dehydration was calculated using the following formula [38]:

PercentDehydration=100%*[(Post‐IllnessWeight–AdmissionWeight)/Post‐IllnessWeight]

Patients were then categorized as having severe (>9%) dehydration, some (3–9%) dehydration, or no (<3%) dehydration based on current standards in the literature [11,14,39].

#### Derivation of clinical diagnostic models

Standard methods, including the Transparent Reporting of a Multivariable Prediction Model for Individual Prognosis or Diagnosis (TRIPOD) guidelines, were used to develop clinical diagnostic models [40–43]. Patients were divided *a priori* into three separate age groups based on the WHO classification of children/adolescents (age 5–19), adults (age 20–59), and elderly (age ≥ 60) [4,44,45]. Forward stepwise regression techniques were used to fit the candidate variables into final ordinal regression models to predict dehydration severity (none, some, or severe) for the population as a whole and separately for each age group. Both a full NIRUDAK model utilizing age, sex, and all 16 clinical predictors listed above and a simplified NIRUDAK model utilizing only the 9 basic clinical predictors were fit. Given the small number of patients missing data on predictors or outcomes, listwise deletion was used instead of multiple imputation for all models.

Categorical predictor variables were modeled using a set of indicator variables relative to a chosen normal reference level. For the full model, the scale of continuous variables (linear or logarithmic) was chosen based on their distribution. Vomiting episodes, diarrheal episodes, and duration of diarrhea were converted to categorical variables due to uneven frequencies. Continuous variables were modeled both on a linear scale and as restricted cubic splines using knots at the 10^th^, 50^th^, and 90^th^ quantiles. Models both with and without interactions were explored. A forward stepwise regression algorithm was used to select the best model size via 10-fold cross validation. Each dataset was treated with kth fold (k = 1,..,10) left out as the training data and the kth fold as testing data. On the training data starting from a null model, the algorithm iterated through all the remaining predictors that augmented the previous model and selected the one with the lowest training mean squared error (MSE). The testing MSE was then computed on the testing dataset. In the end, each dataset yielded p models (p is the total number of candidate predictors) and thus p testing MSE’s. The optimal model size, m, was chosen with the lowest average testing MSE across 10 folds. The final model was then developed by applying forward stepwise regression to the whole data set and stopping when the model had m variables (see S2 Text for additional detail) [40–43].

#### Model assessment and validation

The full and simplified NIRUDAK models and the age-specific NIRUDAK models were examined for adherence to model assumptions and were assessed for their accuracy, including discrimination and calibration, as well as their reliability, for predicting severe dehydration [46–50]. Model discrimination was assessed using the area under the receiver-operator characteristic (ROC) curve (AUC) for the diagnosis of severe dehydration [42,51]. Levels of sensitivity and specificity were identified at points along the ROC curve. Additionally, the m-index was computed using the weighted average of all 6 possible pairwise AUC comparisons for the three ordinal categories of dehydration (i.e. none versus some, some versus none, some versus severe, severe versus some, none versus severe, and severe versus none) to create a single measure of discrimination for each model [52,53]. The m-index is interpreted like the traditional AUC for a binary diagnostic model: 0.5 is no better than chance, while 1 represents a perfect model. Model calibration was assessed by comparing the average predicted number of patients with severe dehydration versus the average observed number of patients with severe dehydration by deciles of predicted risk [46–50]. Calibration in the large was used to compare observed and predicted endpoints by estimating the intercept in the calibration plot with the slope set at 1. To determine the calibration slope, observed and predicted endpoints were compared by estimating both the intercept and slope. An ideal line is described as having an intercept of 0 and slope of 1 [46]. Reliability was assessed by comparing the model prediction of severe dehydration from each nurse’s independent assessment using the Intraclass Correlation Coefficient (ICC) [54]. Bootstrapping (random selection with replacement) with 1000 iterations was used to correct for over-optimism in estimating the m-index of each model (including the WHO algorithm) and to directly compare the m-index of each NIRUDAK model to the WHO algorithm (S2 Text) [40]. All statistical analyses were performed using R Version 3.6.2.

### Sample size

While a general rule for calculating the sample size for the development of a clinical diagnostic model recommends 10 positive events per variable (EPV) considered for the model, other research suggests that 5 EPV is sufficient in most scenarios [41,42,55]. For this study, a minimum of 90 positive outcomes, or 90 patients with each category of dehydration in each age group, was required to achieve 10 EPV for our basic set of 9 clinical predictors (simplified model) and 5 EPV for our expanded set of 18 predictors (full model). Based on an estimated prevalence of severe dehydration in 10% of children/adolescents and 20% of adult/elderly presenting to Dhaka Hospital with acute diarrhea and a 10% loss to study follow-up, an initial target enrollment of 1980 subjects was planned for this study [4,24].

## Results

### Study population characteristics

**Fig 2:**
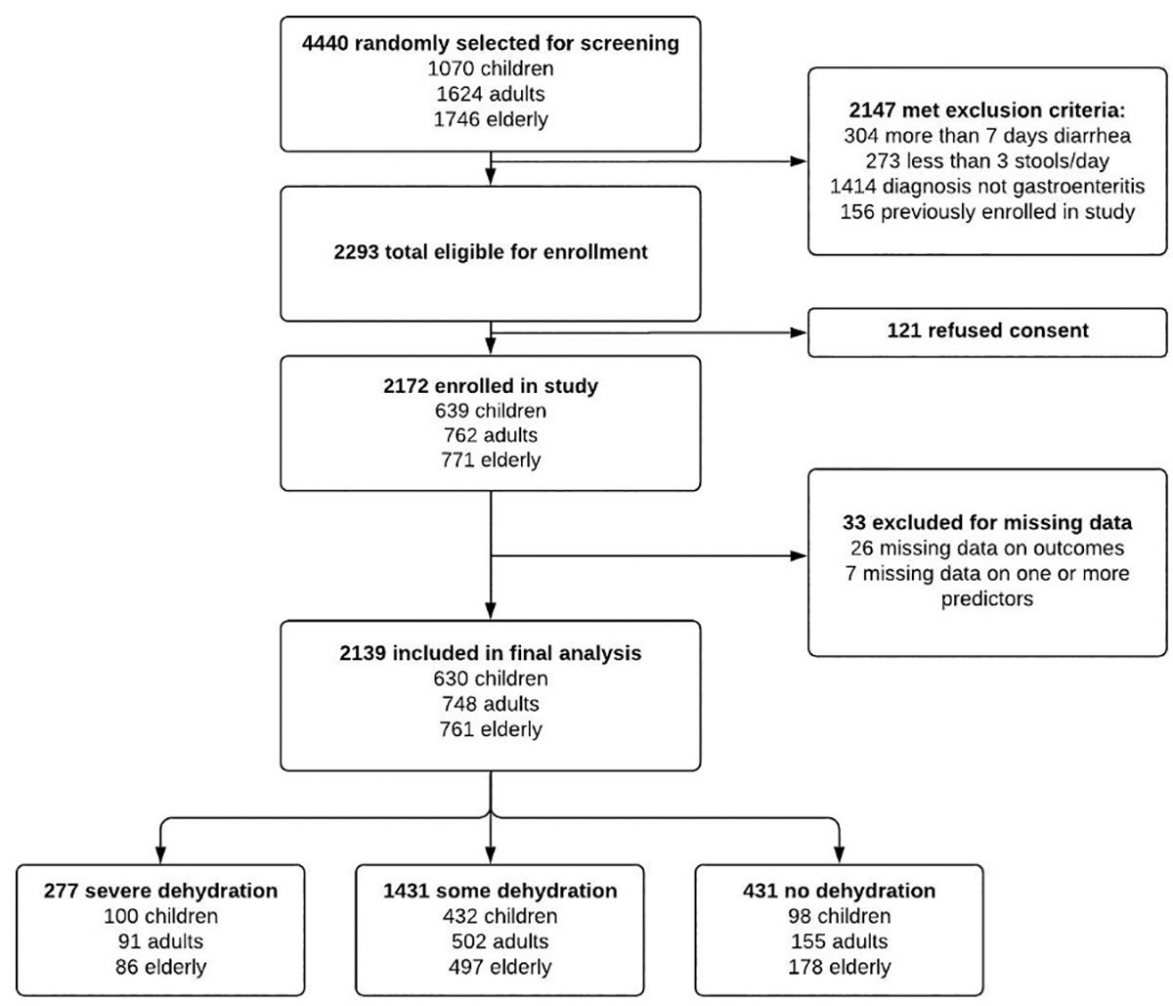
Patient Enrollment.

A total of 4,440 patients over five years presenting to icddr,b with diarrhea were randomly selected for screening, of which 2,293 patients were eligible and 2,172 were enrolled (Fig 2).

Median age for enrolled patients was 35 years (IQR 18–60 years) and 1077 (49.6%) were female. Overall, 278 patients (13%) were classified as having severe dehydration based on our criterion standard, including 100 (16%) children, 91 (12%) adults, and 87 (11%) elderly (Table 1). In addition to age and sex, standardized data were collected for 16 clinical signs and symptoms of dehydration on arrival (Tables 2 and 3). 2039 patients achieved a stable weight prior to discharge with a median time of 18 hours. Of the 133 patients who did not achieve a stable weight prior to discharge, 107 returned to Dhaka Hospital to obtain a final weight within a median time of 3 days, and the remaining 26 were lost to follow up.

**Table 1:** Population Characteristics.

	Overall (N = 2139)	Children (N = 630)	Adults (N = 748)	Elderly (N = 761)
**Sociodemographic Variables**^a^				
Age (years), median (IQR)	35.0(18.0–60.0)	14.0(10.0–17.0)	30.0(25.0–40.0)	62.0(60.0–66.0)
Sex, No. (%)				
Female	1063(49.7)	256(40.6)	409(54.7)	498(52.3)
Male	1095(50.4)	381(59.6)	347(45.5)	367(47.6)
Home location, No. (%)				
Urban	1628(76.1)	507(80.5)	586(78.3)	535(70.3)
Rural/Suburban	511(23.9)	123(19.5)	162(21.7)	226(29.7)
Years of education,^b^ median (IQR)	3.0(0.0–7.0)	5.0(2.0–7.0)	5.0(2.0–9.0)	0.0(0.0–4.0)
Monthly household income (USD), median (IQR)	168.0 (120.0–240.0)	144.0 (120.0–204.0)	168.0 (120.0–240.0)	180.0 (120.0–240.0)
People living in household, median (IQR)	5.0(4.0–6.0)	5.0(4.0–6.0)	4.0(3.0–6.0)	5.0(4.0–6.0)
**Clinical Variables**				
Nutritional status (MUAC), No. (%)				
Severe wasting	31(1.4)	20(3.2)	3(0.4)	8(1.1)
Moderate wasting	164(7.7)	96(15.2)	21(2.8)	47(6.2)
No wasting	1944(90.9)	514(81.6)	724(96.8)	706(92.8)
Enteric Pathogen, No. (%)^c^				
E. coli	834(39.0)	249(39.5)	289(38.6)	296(38.9)
ETEC	212(9.9)	52(8.3)	80(10.7)	80(10.5)
EPEC	5(0.2)	1(0.2)	2(0.3)	2(0.3)
EHEC	0	0	0	0
EIEC	36(1.7)	19(3.0)	6(0.8)	11(1.4)
EAEC	609(28.5)	188(29.8)	209(27.9)	212(27.9)
Vibrio cholera	632(29.5)	260(41.2)	187(25.0)	185(24.3)
Aeromonas	396(18.5)	98(15.6)	160(21.4)	138(18.1)
Campylobacter	219(10.2)	121(19.2)	62(8.3)	36(4.7)
Salmonella	59(2.8)	8(1.3)	26(3.5)	25(3.3)
Shigella	42(2.0)	20(3.2)	7(0.9)	15(2.0)
Other Bacteria^d^	14(0.7)	3(0.5)	7(0.9)	4(0.5)
No Bacteria Detected	620(29.0)	145(23.0)	224(29.9)	251(33.0)
**Outcome**				
Dehydration category, No. (%)^e^				
Severe dehydration	277(12.9)	100(15.9)	91(12.2)	86(11.3)
Some dehydration	1431(66.9)	432(68.6)	502(67.1)	497(65.3)
No dehydration	431(20.1)	98(15.6)	155(20.7)	178(23.4)

a Categorical variables were summarized as percent (n), continuous variables summarized as mean (standard deviation) or median (25^th^, 75^th^ percentile) if not normally distributed

b For patients under 16 years old, years of mother’s education was used

c As detected by PCR (E. coli only) or stool culture (all other bacteria). 37 patients were missing stool samples for culture/PCR. Numbers do not sum to 100% as some patients had more than 1 pathogen detected on stool culture/PCR.

d Other bacteria includes the following species: Plesiomonas shigelloides, Vibrio fluvialis, Vibrio Parahaemolyticus, Vagococcus fluvialis

e 26 patients were missing data on dehydration category

**Table 2:** Clinical Symptoms and Signs on Arrival by Age.

Basic Predictors Assessed for Simplified NIRUDAK Model				
	Overall (N = 2139)	Children (N = 630)	Adults (N = 748)	Elderly (N = 761)
Mental status, No. (%)				
Normal	2046(95.7)	611(97.0)	707(94.5)	728(95.7)
Confused/lethargic	93(4.3)	19(3.0)	41(5.5)	33(4.3)
Thirst, No. (%)				
Normal	177(8.3)	60(9.5)	70(9.4)	47(6.2)
Drinks eagerly	1253(58.6)	377(59.8)	439(58.7)	437(57.4)
Refuses/unable to drink	709(33.1)	193(30.6)	239(32.0)	277(36.4)
Skin pinch, No. (%)				
Rapid	825(38.6)	290(46.0)	374(50.0)	161(21.2)
Slow	1053(49.2)	313(49.7)	310(41.4)	430(56.5)
Very slow	261(12.2)	27(4.3)	64(8.6)	170(22.3)
Eye level, No. (%)				
Normal	546(25.5)	209(33.2)	193(25.8)	144(18.9)
Sunken	1593(74.5)	421(66.8)	555(74.2)	617(81.1)
Mucous membranes, No. (%)				
Moist	49(2.3)	16(2.5)	19(2.5)	14(1.8)
Dry	2090(97.7)	614(97.5)	729(97.5)	747(98.2)
Respiration depth, No. (%)				
Normal	871(40.7)	239(37.9)	363(48.5)	269(35.3)
Deep	1268(59.3)	391(62.1)	385(51.5)	492(64.7)
Radial pulse, No. (%)				
Strong	651(30.4)	170(27.0)	250(33.4)	231(30.4)
Decreased	1443(67.5)	449(71.3)	482(64.4)	512(67.3)
Absent	45(2.1)	11(1.7)	16(2.1)	18(2.4)
Capillary refill, No. (%)				
Normal	2097(98.0)	611(97.0)	739(98.8)	747(98.2)
Prolonged	42(2.0)	19(3.0)	9(1.2)	14(1.8)
Urine output, No. (%)				
Normal	199(9.3)	45(7.1)	84(11.2)	70(9.2)
Decreased/dark	1708(79.9)	519(82.4)	568(75.9)	621(81.6)
Minimal/none	232(10.8)	66(10.5)	96(12.8)	70(9.2)
**Additional Predictors Assessed for Full NIRUDAK Model**				
Vomiting episodes in 24 hours, No. (%)				
None	208(9.7)	26(4.1)	65(8.7)	117(15.4)
1–5	729(34.1)	182(28.9)	259(34.6)	288(37.8)
6–10	770(36.0)	260(41.3)	275(36.8)	235(30.9)
>10	432(20.2)	162(25.7)	149(19.9)	121(15.9)
Diarrhea episodes in 24 hours, No. (%)				
<10	434(20.3)	168(26.7)	143(19.1)	123(16.2)
10–19	986(46.1)	306(48.6)	341(45.6)	339(44.5)
>19	719(33.6)	156(24.8)	264(35.3)	299(39.3)
Duration of diarrhea, No. (%)				
<13 hours	752(35.2)	212(33.7)	283(37.8)	257(33.8)
13–23 hours	328(15.3)	85(13.5)	134(17.9)	091(14.3)
> 23 hours	1059(49.5)	333(52.9)	331(44.3)	395(51.9)
Heart rate, mean (SD)				
Flat	96.8(20.7)	106.3(22.8)	95.9(18.6)	89.9(17.8)
Sitting	103.8(21.5)	114.3(23.5)	103.6(18.6)	95.3(18.3)
Systolic BP, mean (SD)				
Flat	94.2(20.6)	88.2(16.2)	94.2(19.7)	99.2(23.3)
Sitting	94.1(20.8)	88.6(17.1)	94.7(20.2)	98.2(23.1)
Diastolic BP, mean (SD)				
Flat	63.6(15.2)	59.7(13.4)	64.8(15.7)	65.6(15.4)
Sitting	64.7(15.0)	61.2(13.3)	66.4(15.8)	65.9(15.0)
MUAC, mean (SD)	236.4(36.7)	206.3(36.6)	254.6(29.0)	243.4(27.2)

**Table 3:** Clinical Symptoms and Signs on Arrival by Dehydration Category.

Predictors	Overall (N = 2139)	Severe Dehydration (N = 277)	Some Dehydration (N = 1431)	No Dehydration (N = 431)
Sex, No. (%)				
Male	1076 (50.3)	163(58.8)	726 (50.7)	187 (43.4)
Female	1063 (49.7)	114(41.2)	705 (49.3)	244 (56.6)
Mental status, No. (%)				
Normal	2046(95.7)	254(91.7)	1370(95.7)	422(97.9)
Confused/lethargic	93(4.3)	23(8.30)	61(4.3)	9(2.1)
Thirst, No. (%)				
Normal	177(8.3)	14(5.2)	101(7.1)	62(14.4)
Drinks eagerly	1253(58.6)	151(54.5)	842(58.8)	260(60.3)
Refuses/unable to drink	709(33.1)	112(40.4)	288(20.1)	109(25.3)
Skin pinch, No. (%)				
Rapid	825(38.6)	31(11.2)	526(36.8)	268(62.2)
Slow	1053(49.2)	163(58.8)	746(52.1)	144(33.4)
Very slow	261(12.2)	83(30.0)	159(11.1)	19(4.4)
Eye level, No. (%)				
Normal	546(25.5)	30(10.8)	318(22.2)	198(45.9)
Sunken	1593(74.5)	247(89.2)	1113(77.8)	233(54.1)
Mucous membranes, No. (%)				
Moist	49(2.3)	4(1.4)	26(1.8)	19(4.4)
Dry	2090(97.7)	273(98.6)	1405(98.2)	412(95.6)
Respiration depth, No. (%)				
Normal	871(40.7)	53(19.1)	551(38.5)	267(61.9)
Deep	1268(59.3)	224(80.9)	880(61.5)	164(38.1)
Radial pulse, No. (%)				
Strong	651(30.4)	43(15.5)	389(27.2)	219(50.8)
Decreased	1443(67.5)	220(79.4)	1012(70.1)	211(49.0)
Absent	45(2.1)	14(5.05)	30(2.1)	1(0.2)
Capillary refill, No. (%)				
Normal	2097(98.0)	269(97.1)	1401(97.9)	427(99.1)
Prolonged	42(2.0)	8(2.9)	30(2.1)	4(0.9)
Urine output, No. (%)				
Normal	199(9.3)	16(5.8)	108(7.5)	75(17.4)
Decreased/dark	1708(79.9)	215(77.6)	1166(81.4)	327(75.9)
Minimal/none	232(10.8)	46(16.6)	157(11.0)	29(6.7)
Vomiting episodes in 24 hours, No. (%)				
None	208(9.7)	11(4.0)	115(8.0)	82(19.0)
1–5	936(34.1)	111(40.1)	619(43.3)	206(47.8)
6–10	802(36.0)	122(44.0)	552(38.6)	128(29.7)
>10	193(20.2)	33(11.9)	145(10.1)	15(3.5)
Diarrhea episodes in 24 hours, No. (%)				
<10	434(20.3)	62(22.4)	260(18.2)	112(26.0)
10–19	986(46.1)	110(39.7)	677(47.3)	199(46.2)
>19	719(33.6)	105(37.9)	494(34.5)	120(27.8)
Duration of diarrhea, No. (%)				
<13 hours	752(35.2)	109(39.4)	509(35.6)	134(31.1)
13–23 hours	328(15.3)	37(13.4)	223(15.6)	68(15.8)
> 23 hours	1059(49.5)	131(47.3)	699(48.8)	229(53.1)
Heart rate, mean (SD)				
Flat	96.8(20.7)	97.8(23.1)	96.9(20.6)	96.1(19.6)
Sitting	103.8(21.5)	105.9(23.4)	103.5(21.5)	103.4(19.7)
Systolic BP, mean (SD)				
Flat	94.2(20.6)	84.6(17.8)	92.9(19.4)	104.84(22.1)
Sitting	94.1(20.8)	84.6(18.4)	92.6(19.4)	105.2(22.3)
Diastolic BP, mean (SD)				
Flat	63.6(15.2)	57.3(14.0)	63.2(15.1)	68.9(14.1)
Sitting	64.7(15.0)	59.3(13.8)	64.0(14.7)	70.8(15.0)
MUAC, mean (SD)	236.4(36.7)	248.3(39.5)	237.0(34.7)	214.7(32.6)

#### Development of Ordinal Regression Models to Predict Dehydration

After excluding 33 (1.5%) subjects missing data on either dehydration predictors or the final outcome (7 (0.3%) missing blood pressure and 26 (1.2%) missing percent dehydration), 2,139 were included in the development of the final diagnostic models, including 630 children, 748 adults, and 761 elderly patients (Fig 2). Neither restricted cubic splines nor interaction terms improved model performance, so were not included in the final models. Tables 4 and 5 summarizes the variables selected and intercepts for the age specific and final full and simplified NIRUDAK models, respectively, alongside their regression coefficients and odds ratios. Note that the odds ratios listed represent both the odds ratio for predicting any dehydration compared to no dehydration and the odds ratio for predicting severe dehydration compared to no severe dehydration. For the full NIRUDAK model, the odds of any dehydration were 2.03 times greater if the patient had slow skin pinch compared to rapid skin pinch, and similarly the odds of severe dehydration were also 2.03 times greater for slow skin pinch compared to rapid skin pinch. The odds of any dehydration were 4.6 times greater if the patient had very slow skin pinch compared to rapid skin pinch and the odds of severe dehydration were also 4.6 times greater for very slow skin pinch compared to rapid skin pinch.

### Age-specific NIRUDAK model performance

We derived age-specific models for the diagnosis of dehydration category for patients under age 20, age 20–59, and over age 60. Table 4 summarizes the variables selected for each age-specific model and their odds ratios. While skin pinch and MUAC were selected in all three age groups, other signs/symptoms were only selected for 1 or 2 age groups. The m-index for the age-specific NIRUDAK models derived separately for children, adults, and elderly was 0.75, 0.78, and 0.76, respectively. The calibration intercept for the children’s age group was -0.011 (p = 0.92) and the calibration slope was 1.15 (p = 0.83). For the adult age group, the calibration intercept was -0.017 (p = 0.89) and the calibration slope was 1.11 (p = 0.80). For the elderly age group, the calibration intercept was -0.026 (p = 0.83) and the calibration slope was 1.22 (p = 0.95). Discrimination, measured using the area under the ROC curve for predicting severe dehydration, was 0.77 (95% CI: 0.77, 0.82) for children, 0.81 (95% CI: 0.77, 0.86) for adults, and 0.82 (95% CI: 0.77, 0.86) for the elderly.

**Table 4:** Age-Specific NIRUDAK Model.

	Children/Adolescents OR (95% CI)	Adults OR (95% CI)	Elderly OR (95% CI)
Skin pinch			
Rapid (Reference Level)	1	1	1
Slow	2.28(1.49,3.53)	2.65(1.74,4.1)	1.92(1.29,2.87)
Very slow	2.24(0.88,5.61)	5.56(2.85,10.92)	4.85(2.79,8.51)
Eye level			
Normal (Reference Level)	1	1	-
Sunken	2.28(1.49,3.5)	2.3(1.57,3.39)	-
Respiration depth			
Normal (Reference Level)	1	-	1
Deep	1.47(0.98,2.21)	-	1.67(1.18,2.37)
Vomiting episodes in 24 hours			
<1 (Reference Level)	1	-	1
1–5	4.67(1.94,11.7)	-	1.78(1.11,2.83)
6–10	9.94(4.15,24.78)	-	1.73(1.05,2.83)
>10	11.88(4.79,30.67)	-	2.45(1.38,4.36)
Diarrheal episodes in 24 hours			
<10 (Reference Level)	1	-	-
10–19	0.70(0.45,1.07)	-	-
>19	0.87(0.51,1.48)	-	-
Duration of diarrhea			
<13 hours (Reference Level)	1	-	-
13–23 hours	0.53(0.3,0.94)	-	-
>23 hours	1.05(0.7,1.58)	-	-
HR difference^a^ (per 10 beat per minute increase)	1.12(0.98,1.29)	-	-
Diastolic BP difference^a^ (per 10 mmHg increase)	0.80(0.66,0.98)	-	-
Systolic BP laying down (per 10 mmHg increase)	-	0.8(0.77,0.92)	0.85(0.79,0.91)
MUAC (per 10 mm increase)	0.94(0.89,0.99)	0.83(0.78,0.88)	0.86(0.81,0.92)
Sex			
Female (Reference Level)	-	-	1
Male	-	-	1.63(1.18,2.25)

a Difference between the measurements when the patient is sitting up versus lying flat.

- signifies that the predictor was not included in the model for that age group.

1 signifies reference category for the variable

### Full and simplified NIRUDAK model performance

**Fig 3:**
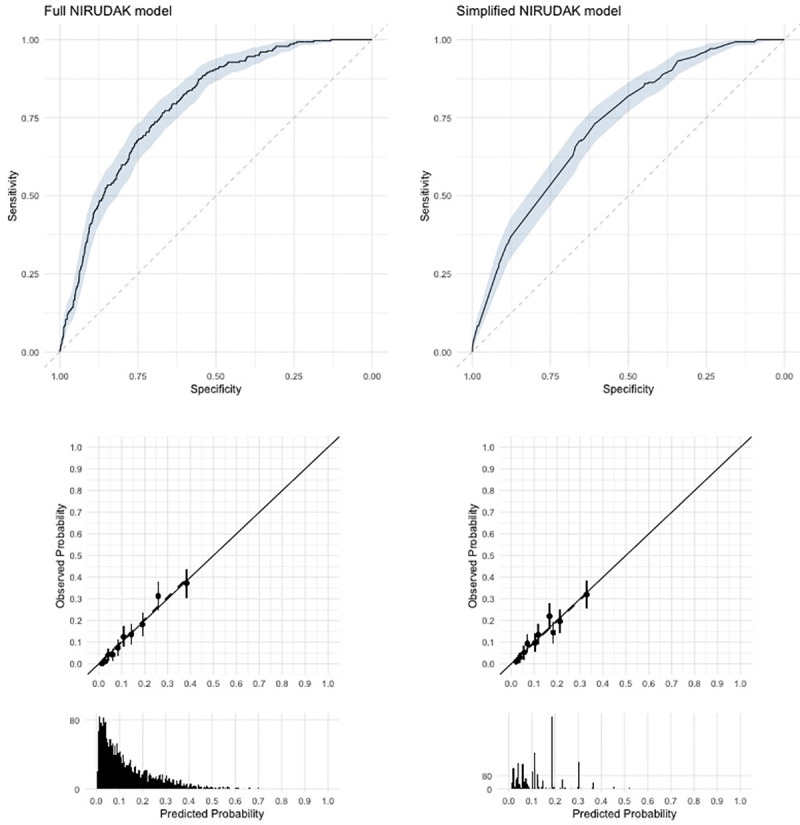
Discrimination and calibration for NIRUDAK models based on training data. The top row shows Receiver Operating Characteristic Curves. Shaded area gives 95% confidence regions. The middle row plots observed versus predicted by deciles of predicted probabilities. The vertical lines give the 95% confidence intervals for the observed probability in each decile. The bottom row gives histograms of the predicted probabilities.

We derived the Full NIRUDAK model using all 16 clinical predictors along with age and sex for the entire study population, as well as a simplified model using on the 9 basic clinical predictors (Table 5). Fig 3 demonstrates the calibration plot for the full and simplified NIRUDAK models. The calibration intercept for the full NIRUDAK model was -0.014 (p = 0.84) and the calibration slope was 1.11 (p = 0.92). For the simplified NIRUDAK model, the calibration intercept was -0.004 (p = 0.95) and the calibration slope was 1.05 (p = 0.72). Discrimination, measured using the area under the ROC curve for predicting severe dehydration (Fig 3), was 0.79 (95% CI: 0.76–0.82) for the full model and 0.73 (95% CI: 0.70, 0.76) for the simplified model. The full NIRUDAK model achieved a sensitivity of 80% with a specificity of 63% using a cut-off of 0.115 for the probability of severe dehydration, while the simplified NIRUDAK model achieved a sensitivity of 80% with a specificity of 58% using a cut-off of 0.108 for the probability of severe dehydration. Reliability was assessed by comparing the models performed by each nurse’s individual assessments. As measured by the ICC, reliability was 0.90 (95% CI = 0.87, 0.91) for the full model and 0.82 (95% CI = 0.77, 0.86) for the simplified model.

**Table 5:** Full and Simplified NIRUDAK Models.

	Full NIRUDAK Model		Simplified NIRUDAK Model	
	Regression Coefficients	OR (95% CI)	Regression Coefficients	OR (95% CI)
Age (per 1 year increase)	-0.01	0.99(0.99,1.00)	-	-
Skin Pinch				
Rapid (Reference Level)	1	1	1	1
Slow	0.71	2.03(1.60,2.58)	0.68	1.98(1.58,2.49)
Very Slow	1.53	4.60(3.15,6.74)	1.34	3.83(2.72,5.40)
Eye Level				
Normal (Reference Level)	1	1	1	1
Sunken	0.70	2.02(1.61,2.55)	0.60	1.82(1.46,2.29)
Respiration Depth				
Normal (Reference Level)	1	1	1	1
Deep	0.37	1.45(1.17,1.79)	0.58	1.79(1.45,2.20)
Vomiting Episodes in 24 hours			-	-
<1 (Reference Level)	1	1		
1–5	0.39	1.48(1.07,2.07)		
6–10	0.69	1.99(1.42,2.79)		
>10	0.91	2.48(1.71,3.60)		
Systolic BP Flat (per 10 mmHg increase)	-0.14	0.87(0.82,0.91)	-	-
MUAC (per 10 mm increase)	-0.09	0.92(0.89,0.94)	-	-
Sex			-	-
Female (Reference Level)	1	1		
Male	0.36	1.43(1.19,1.73)		
Urine Output	-	-		
Normal (Reference Level)			1	1
Decreased/Dark			0.29	1.33(0.97,1.82)
Minimal/None			0.58	1.78(1.18,2.69)
Radial Pulse	-	-		
Strong (Reference Level)			1	1
Decreased			0.44	1.55(1.25,1.93)
Absent			1.07	2.91(1.53,5.49)
Intercept				
Any Dehydration	-3.38	0.03	0.29	1.34
Severe Dehydration	0.66	1.94	4.08	59.09

- signifies that the predictor was not included in the model for that age group.

1 signifies reference category for the variable

### Model validation and comparison to WHO IMAI algorithm

The m-index for the full NIRUDAK model was 0.75 in the original dataset, while its average optimism corrected performance across 1000 bootstrap iterations was 0.74 (95% CI = 0.72, 0.76). The m-index for the simplified NIRUDAK model was 0.71 in the original dataset, while its average optimism corrected performance was 0.71 (95% CI = 0.69, 0.73).

**Fig 4:**
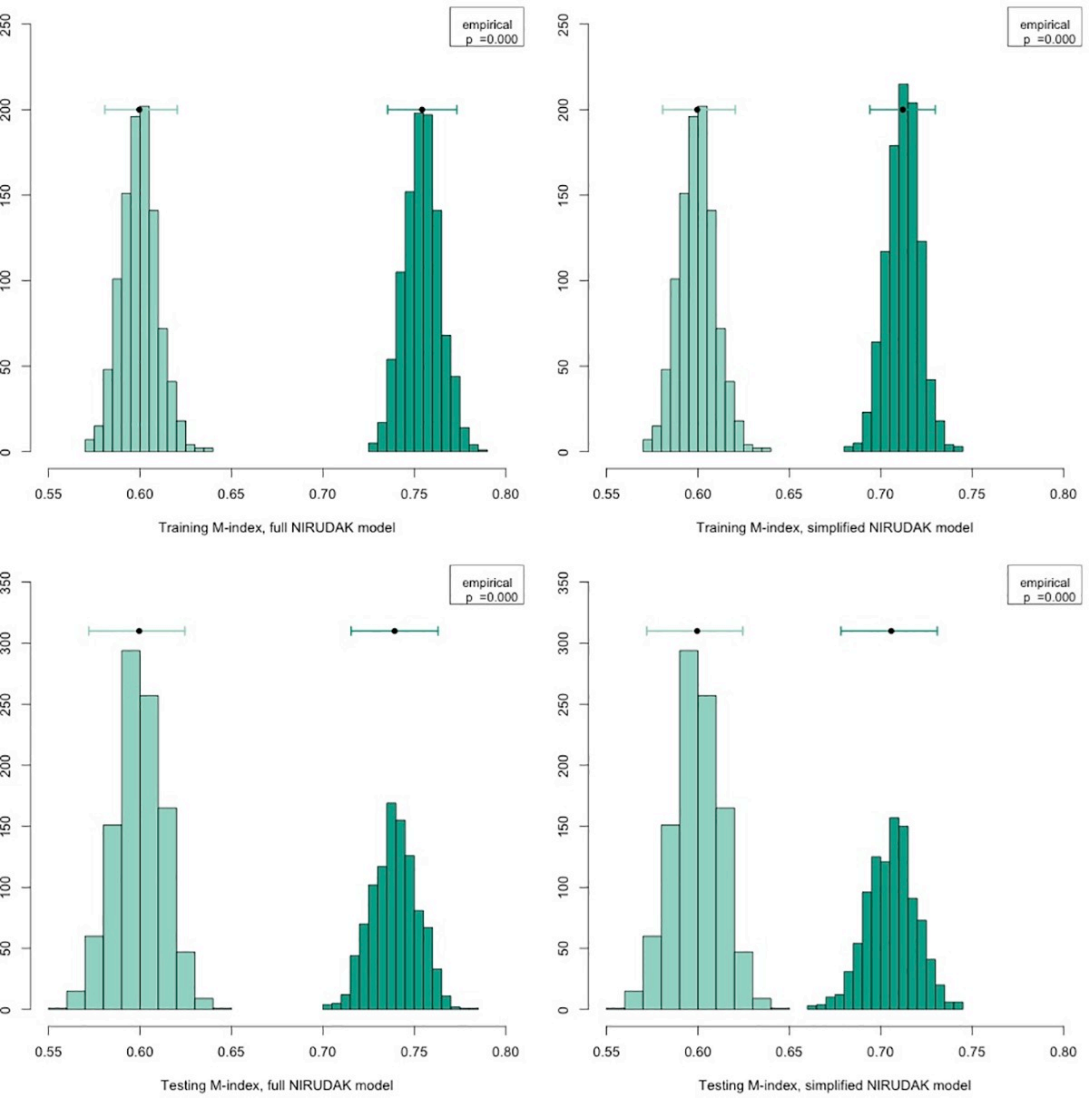
**Comparison of m-indices computed from NIRUDAK full and simplified models (dark green) and WHO IMAI algorithm (light green).** The m-index for each model is computed both from those observations from the original dataset that were included in each bootstrap sample (training m-index) and from those observations excluded from each set (testing m-index). Each histogram shows the distribution of m-indices derived from the bootstrap samples. Histograms for the WHO IMAI algorithm are the same in the left and right columns of the figures. The bar at the top of each histogram gives the mean m-index and a 95 percent confidence interval derived from the bootstraps.

We compared the NIRUDAK models’ performance, as measured using the m-index, to the WHO algorithm [10]. As shown in Fig 4 below, the m-index for the full and simplified NIRUDAK models were significantly better than those of the WHO algorithm, both in the bootstrap training and testing datasets (p<0.001 for all comparisons).

The WHO algorithm’s specificity for detecting severe dehydration was 69% in our population, while its sensitivity was only 53%. At the same level of 69% specificity, the full NIRUDAK model had a sensitivity of 74% while the simplified NIRUDAK model had a sensitivity of 63%.

## Discussion

NIRUDAK is the first study to empirically derive clinical diagnostic models for assessing dehydration severity in patients over 5 years with acute diarrhea. The NIRUDAK models derived in this study were found to be accurate, with both good discrimination and calibration, as well as reliable, based on standards in the literature [40–43,51,54]. All models showed only minimal optimism, suggesting they will continue to perform well in new populations of patients. Additionally, a single combined model performed as well as age-specific models, simplifying usage in practice.

To determine the relative utility of our newly developed models, we compared them directly to the WHO algorithm for dehydration assessment in adults and adolescents with acute diarrhea, considered the standard of care for diarrhea management in most low resource settings. Unlike the NIRUDAK models, the WHO algorithm was never empirically derived for use in this population, but was rather adapted from the prior IMCI algorithm for dehydration assessment, which itself had been developed based on expert consensus [10,13].

Our NIRUDAK models significantly outperformed the WHO algorithm, both in the bootstrap training datasets (which may be biased towards our models) and the bootstrap testing datasets (which should allow for a fair comparison). While the specificity of the WHO algorithm for detecting severe dehydration was moderate, its sensitivity was quite poor. This may be explained by differences in both the etiology of diarrhea and physiologic responses to dehydration in young children (for which the WHO algorithm was originally designed) when compared to older patients. For instance, older patients are more likely to have bacterial causes of diarrhea, and changes in mental status or thirst (major components of the WHO algorithm) may be less pronounced in older patients [6,21–23]. For the same level of specificity as the WHO algorithm, our NIRUDAK models provide much better sensitivity for severe dehydration, reducing the likelihood of under-triage and under-treatment of these high-risk patients. This distinction between severe dehydration and some dehydration is especially important, as patients with severe dehydration will require referral to a hospital and the administration of intravenous fluids, while patients with some dehydration can be managed in the community setting with ORS alone [12].

### Limitations

This study was conducted at a single center whose patient population may not be representative of all patients over five years with diarrhea worldwide. Prior research, however, has found the most common causes of diarrhea in patients over five years at icddr,b to be similar to those for patients in other low resource settings, including cholera, Enterotoxigenic E. coli, and shigella [4,21,24,56]. In addition, the reputation of icddr,b and its free services attract a diverse array of patients from a catchment area of nearly 17 million people, including patients from urban, suburban, and rural settings [24]. To improve generalizability, we specifically chose research nurses from outside of icddr,b to collect data for this study, whose experience levels would be more representative of nurses in other low resource settings.

Patients with acute diarrhea presenting for medical care are likely to be more dehydrated on average than patients with acute diarrhea who do not seek care. However, this should not affect the accuracy of our models, given that we enrolled adequate numbers of patients in each dehydration category based on our initial sample size estimates to derive stable models. Some of the included predictors were based on patient recall and may be subject to bias. Finally, the models developed for this study did not include any biomarkers, which may have improved their accuracy, but would have limited their utility in low resource settings, where most individuals with acute diarrhea are managed worldwide [35–37,57].

While the full NIRUDAK model is more complex than the WHO algorithm, requiring clinicians to assess blood pressure and MUAC, our simplified NIRUDAK model is similar in complexity to the WHO algorithm and still performs better. Incorporation of both these NIRUDAK models into a mobile phone (mHealth) application and development of a simple numerical score are currently underway, and will further simplify their use in clinical practice.

## Conclusion

NIRUDAK is the first study to empirically derive clinical diagnostic models for the assessment of dehydration severity in patients over five years of age. These models were found to be accurate and reliable in the population studied, and outperformed the WHO algorithm for dehydration assessment. Use of the NIRUDAK models instead of the current WHO algorithm could result in a significant reduction in under-triage and under-treatment of patients over five years with severe dehydration due to acute diarrhea, potentially reducing the current mortality of one million deaths per year in this population. Currently, additional qualitative research is being conducted to understand the feasibility and acceptability of diagnostic models by providers. After incorporation of the NIRUDAK models into a mHealth application or simple numerical score, they should be externally validated in a new population of patients over five years with acute diarrhea prior to recommendation for use in clinical practice.

## Supporting information

S1 Text Pre-defined protocols for measurement of all clinical variables. (DOCX)

S2 Text Protocols for derivation and internal validation of all models. (DOCX)
