# Correction: Derivation of the first clinical diagnostic models for dehydration severity in patients over five years with acute diarrhea

**DOI:** 10.1371/journal.pntd.0011026

**Published:** 2022-12-30

**Authors:** Adam C. Levine, Meagan A. Barry, Monique Gainey, Sabiha Nasrin, Kexin Qu, Christopher H. Schmid, Eric J. Nelson, Stephanie C. Garbern, Mahmuda Monjory, Rochelle Rosen, Nur H. Alam

There are errors in Table 5. The reference levels for regression coefficients should be 0 instead of 1. The cutoff levels for vomiting in the left-most column should be 1–4, 5–9, and >9 instead of 1–5, 6–10, and >10.

**Table 5:** Full and Simplified NIRUDAK Models.

	Full NIRUDAK Model		Simplified NIRUDAK Model	
	Regression Coefficients	OR (95% CI)	Regression Coefficients	OR (95% CI)
Age (per 1 year increase)	-0.01	0.99(0.99,1.00)	-	-
Skin Pinch				
Rapid (Reference Level)	0	1	0	1
Slow	0.71	2.03(1.60,2.58)	0.68	1.98(1.58,2.49)
Very Slow	1.53	4.60(3.15,6.74)	1.34	3.83(2.72,5.40)
Eye Level				
Normal (Reference Level)	0	1	0	1
Sunken	0.70	2.02(1.61,2.55)	0.60	1.82(1.46,2.29)
Respiration Depth				
Normal (Reference Level)	0	1	0	1
Deep	0.37	1.45(1.17,1.79)	0.58	1.79(1.45,2.20)
Vomiting Episodes in 24 hours			-	-
<1 (Reference Level)	0	1		
1–4	0.39	1.48(1.07,2.07)		
5–9	0.69	1.99(1.42,2.79)		
>9	0.91	2.48(1.71,3.60)		
Systolic BP Flat (per 10 mmHg increase)	-0.14	0.87(0.82,0.91)	-	-
MUAC (per 10 mm increase)	-0.09	0.92(0.89,0.94)	-	-
Sex			-	-
Female (Reference Level)	0	1		
Male	0.36	1.43(1.19,1.73)		
Urine Output	-	-		
Normal (Reference Level)			0	1
Decreased/Dark			0.29	1.33(0.97,1.82)
Minimal/None			0.58	1.78(1.18,2.69)
Radial Pulse	-	-		
Strong (Reference Level)			0	1
Decreased			0.44	1.55(1.25,1.93)
Absent			1.07	2.91(1.53,5.49)
Intercept				
Any Dehydration	-3.38	0.03	0.29	1.34
Severe Dehydration	0.66	1.94	4.08	59.09

- signifies that the predictor was not included in the model for that age group.

1 signifies reference category for the variable
