# Supplementary material for: Derivation of the first clinical diagnostic models for dehydration severity in patients over five years with acute diarrhea
Source: PLoS Negl Trop Dis. 2021 Mar 10;15(3):e0009266. doi: 10.1371/journal.pntd.0009266 (PMC7984611; doi:10.1371/journal.pntd.0009266)
Supplement: S1 Text — (DOCX) [file pntd.0009266.s001.docx]

**S1 Appendix. Pre-defined protocols for measurement of all clinical variables.**

Mental Status

Mental status was assessed by observing and interacting with the patient. If the patient was awake and able to respond appropriately to questions and commands, the mental status was classified as “Normal.” If the patient’s eyes were closed, or the patient was staring into space, or the patient was slow to respond to questions or commands, the patient’s mental status was classified as “Confused/Lethargic”.

Thirst

Thirst was evaluated by pouring a small amount of water into a cup and offering it to the patient. The patient’s thirst was classified as “Normal” if the patient sipped the water slowly or “Drinks Eagerly” if they drank it quickly. The patient’s thirst was classified as “Refuses/Unable to Drink” if they refused or were unable to drink water.

Skin Pinch

A skin pinch test was performed on the patient by grasping a fold of skin on the side of their abdomen between the thumb and index finger and rapidly releasing the skin while counting how many seconds it took for the skin to flatten again. “Rapid” was defined by the skin flattening immediately (in the blink of an eye). “Slow” was defined by the skin flattening in about one second. “Very Slow” was defined by the skin flattening in two or more seconds.

Eye Level

The patient’s eye level was evaluated by viewing the patient’s face from the side of the stretcher at the level of the patient and identifying whether the patient’s eyelid was below their orbital rim with their eyes closed. If so, their eye level was classified as “Sunken”, otherwise it was classified as “Normal.” If it was unclear based on visualization, nurses were instructed to place the lateral aspect of one finger across the patient’s orbital rim, with their finger touching both the superior and inferior portions of their orbital rim while the patient’s eyes were closed. The eye level was classified as “Normal” when the nurse could feel the eyelid touching their finger and “Sunken” when the eyelid was below the level of the orbital rim and not touching their finger.

Mucous Membranes

The patient’s mucous membranes were evaluated by asking the patient to open their mouth and observing the oral cavity. When the patient’s lips appeared normal and saliva was clearly visible on or around the tongue, the patient’s mucous membranes were classified as “Normal.” When their lips appeared dry or there was little or no moisture on or around the tongue, their mucous membranes were classified as “Dry.”

Respiration Depth

Respiration depth was evaluated by observing the patient’s abdomen while lying flat. If their skin did not sink below the level of their lower ribs at any point during the respiratory cycle, their respiration depth was classified as “Normal.” If their skin did sink below the level of the lower ribs at any point during the respiratory cycle, their respiration depth was classified as “Deep.”

Radial Pulse

Radial pulse was evaluated by placing two fingers just proximal to the patient’s wrist crease on the radial side of the forearm and comparing the patient’s radial pulse to one’s own. If they were similar, the patient’s radial pulse was classified as “Strong.” If the patient’s radial pulse felt weaker, it was classified as “Decreased.” When the patient’s radial pulse could not be felt at all, it was classified as “Absent.”

Capillary Refill

Capillary refill was evaluated by pressing on the edge of the nailbed of the patient’s thumb, making note of the time for the color to return once pressure was released. If the patient had nail polish, the pad of their thumb was used instead. If the color returned in 2 seconds or less, the capillary refill was classified as “Normal.” If it took longer than 2 seconds to return, the capillary refill was classified as “Prolonged.”

Urine Output

Urine output was evaluated by asking the patient or their family about their urination in the last 8 hours. If the patient felt their urine output was normal for them over this time period, it was classified as “Normal.” If the patient felt their urination was less frequent or darker (more concentrated) than normal over this time period, their urine output was classified as “Decreased/Dark.” If the patient had not urinated at all in the past 8 hours or only a few drops, their urine output was classified as “Minimal/None.”

Vomiting Episodes in 24 hours

Vomiting episodes in 24 hours were assessed by asking the patient or their family member how many discrete episodes of vomiting the patient had within the past 24 hours of presentation.

Diarrheal Episodes in 24 hours

Diarrheal episodes in 24 hours were assessed by asking the patient or their family member how many discrete episodes of diarrhea the patient had within the past 24 hours of presentation.

Duration of Diarrhea

Duration of diarrhea was assessed by asking the patient or their family member how long ago the patient began experiencing diarrhea at presentation.

Heart Rate

Heart rate was assessed by placing a pulse oximeter on the patient’s finger to measure their heart rate while lying flat. If the pulse oximeter was unable to measure heart rate, then the nurse listened to the heart beat with a stethoscope and counted the number of beats over 60 seconds using a stopwatch. A second heart rate measurement was taken while the patient was sitting up by elevating the head of the stretcher to 90 degrees (with a 30 second delay between obtaining the flat and seated measurements to allow time for the heart rate to adjust). The heart rate difference was calculated as the seated heart rate minus the flat heart rate. Standing heart rate was not assessed as many patients were unable to stand due to the severity of illness.

Systolic/Diastolic Blood Pressure

The patient’s blood pressure was obtained while the patient was lying flat using an automated blood pressure cuff. If the patient was receiving IV fluids, the arm opposite of the IV line was used so as to not interfere with treatment. If the automatic blood pressure cuff was not able to obtain a measurement on the first try, a manual cuff was used instead. For children, a manual, child-sized blood pressure cuff was used to measure blood pressure. A second blood pressure measurement was taken while the patient was sitting up by elevating the head of the stretcher to 90 degrees (with a 30 second delay between obtaining the flat and seated measurements to allow time for the heart rate to adjust). The blood pressure difference was calculated as the seated blood pressure minus the flat blood pressure. Standing blood pressure was not assessed as many patients were unable to stand due to the severity of illness.

MUAC

The mid-upper arm circumference (MUAC) was assessed by bending the patient’s left elbow to 90 degrees while their left arm was hanging loosely at their side (not stretched out) and measuring the midpoint between the tip of the shoulder and the tip of the elbow. A standard MUAC tape was wrapped around the arm at the measured midpoint, and the observed number was recorded in millimeters.
