# Supplementary material for: Derivation of the first clinical diagnostic models for dehydration severity in patients over five years with acute diarrhea
Source: PLoS Negl Trop Dis. 2021 Mar 10;15(3):e0009266. doi: 10.1371/journal.pntd.0009266 (PMC7984611; doi:10.1371/journal.pntd.0009266)
Supplement: S2 Text — (DOCX) [file pntd.0009266.s002.docx]

**S2 Appendix: Protocols for derivation and internal validation of all models.**

Forward Stepwise Selection Algorithm for Main Effects via Cross-Validation

1. Divide the data randomly into K equally sized sets. For k in 1,…,K
   1. Treat set k as the holdout test set and the other K-1 sets as the training data
   2. Construct the null model, M_k0_, which contains no predictors and compute model evaluation index *E*_k,0_ (AIC for ordinal models, mean squared error for continuous models)
   3. Assume number of predictors is p. For j = 0, …, p-1:
      1. Consider all p − j models that augment the *j* predictors in *M*_kj_ with one additional predictor; compute model evaluation index for each of p-j models fit to the training data
      2. Choose the best among these p − j models, and call it *M*_k,j+1_
      3. Apply model *M*_k,j+1_ on the holdout test set, and compute model evaluation index *E*_k,j+1_
2. Compute average test performance $\bar{E}_{j}$ for j = 0, 1, …, p across K test sets as $\bar{E}_{j}=\frac{1}{K}\sum_{k=1}^{K} E_{kj}$ and choose m= $\underset{j}{\mathrm{argmin}} \bar{E}_{j}$ as the optimal model size
3. Choose the final model as the result of forward stepwise regression on the whole data set stopping when the model has *m* variables.

Forward Stepwise Selection Algorithm for Interactions via Cross-Validation

Interaction terms were selected by applying the forward stepwise procedure below for all possible pairwise interaction terms after running the main effects algorithm above.

1. Use the same division of k data sets. For k in 1,…,K
   1. Treat set k as the holdout test set and the other K-1 sets as the training data
   2. Take model *M*_k,j_ , *j=2,…,p* obtained from the previous algorithm as the null model with no interactions and define *M*_k,j,0_ = *M*_k,j_. For each j, q_j_ = $\left( \begin{matrix} j \\ 2 \end{matrix} \right)$ potential pairwise interactions are considered
   3. For i = 1, …, q_j_
      1. Consider all q_j_ – i+1 models that augment the j + i-1 predictors (j main effects + i-1 interactions) in *M*_k,j,,i-1_ with one additional interaction pair; compute the model evaluation index for each of q_j_ – i+1 models
      2. Choose the best model among these q_j_ – i+1 models, and call it *M*_k,j,i_
      3. Apply model *M*_k,j,I_ on the holdout test set, and compute model evaluation index *E*_k,j,i_
2. Compute average test performance $\bar{E}_{ji}$ across K test sets as $\bar{E}_{ji}=\frac{1}{K}\sum_{k=1}^{K} E_{kji}$ for each j= 2,…,p and i= 1,…,q_j,_ and choose (m,n) = $\underset{j}{\mathrm{argmin}} \bar{E}_{ji}$ as optimal model size where *m*  is the number of main effects and *n* is the number of interactions in the model.
3. Choose final model as result of forward stepwise regression on whole data set stopping when the model has *m* variables and then *n* interactions among these *j* variables.

Model Validation Algorithm Using Bootstrap

To correct for overoptimism of the m-index measured on the training data, we constructed 1000 bootstrap samples with replacement from the original data and applied the algorithm below to estimate the amount by which the m-index was inflated in the original sample. This inflation factor was then subtracted from the apparent m-index to obtain a corrected m-index.

1. Compute the apparent measure *C_app_* on the full dataset S of size *N_S_* for the final model derived in the model derivation step
2. Generate B=1000 datasets of size *N_S_* from the full data using bootstrap samples with replacement
3. On each of new datasets *b* = 1, 2, …, B, develop a new model *M_b_* using the optimal value of the tuning parameter found in the training model derivation
4. Compute the measure *C_boot,b_* on the bootstrap dataset *b*
5. Compute *C_nboot,b_* using model *M_b_* applied to the *N_S_* -*b* observations left out of bootstrap set b
6. Compute the optimism estimate of each bootstrap sample $O_{b} =C_{boot,b}-C_{nboot,b}$
7. Compute the over-optimism of the original model as $O=\frac{1}{B}\sum_{b=1}^{B} O_{b}$ giving the optimism corrected performance of the original model as *C_adj_ = C_app_ - O_b_*

Comparison of NIRUDAK Models to WHO IMAI Algorithm

Because the WHO algorithm when applied to each individual is deterministic, it returns a predicted category rather than a predicted probability of being in a category. We set the probability of the predicted category to 1 and the probabilities of the other two categories to 0. The m-index was then calculated using the same procedure as for the NIRUDAK models above. The optimism-corrected m-index for the WHO algorithm was then calculated from the 1000 bootstrap samples used for the NIRUDAK models above by the same procedure.

To compare the WHO and NIRUDAK models, denote the M-index computed on each bootstrap dataset *b* and its complement (the observations left out of the bootstrap dataset) by *C^WHO^_boot,b_* and *C^WHO^_nboot,b_* ,respectively, for the WHO model, and by *C^NIRUDAK^_boot,b_* and *C^NIRUDAK^_nboot,b_*, respectively, for the NIRUDAK model. P-value for the comparison were calculated as the proportion where *C^WHO^_boot,b_ < C^NIRUDAK^_boot,b,_ and as the proportion where C^WHO^_nboot,b_ < C^NIRUDAK^_nboot,b_.*
